# Role of *HMOX1* Promoter Genetic Variants in Chemoresistance and Chemotherapy Induced Neutropenia in Children with Acute Lymphoblastic Leukemia

**DOI:** 10.3390/ijms22030988

**Published:** 2021-01-20

**Authors:** Karolina Bukowska-Strakova, Joanna Włodek, Ewelina Pitera, Magdalena Kozakowska, Anna Konturek-Cieśla, Maciej Cieśla, Monika Gońka, Witold Nowak, Aleksandra Wieczorek, Katarzyna Pawińska-Wąsikowska, Alicja Józkowicz, Maciej Siedlar

**Affiliations:** 1 Department of Clinical Immunology, Institute of Pediatrics, Jagiellonian University Medical College, 31-663 Kraków, Poland; asia.wlodek91@gmail.com (J.W.); ewelina.pitera@uj.edu.pl (E.P.); 2 Department of Medical Biotechnology, Faculty of Biochemistry, Biophysics and Biotechnology, Jagiellonian University, 31-007 Kraków, Poland; magda100pa@interia.pl (M.K.); a.konturek@gmail.com (A.K.-C.); maciej.ciesla@med.lu.se (M.C.); monika.gonka@gmail.com (M.G.); witold.nowak@uj.edu.pl (W.N.); 3 Pediatric, Oncology and Hematology Department, Institute of Pediatrics, Jagiellonian University Medical College, 30-387 Krakow, Poland; a.wieczorek@uj.edu.pl (A.W.); katarzyna.pawinska-wasikowska@uj.edu.pl (K.P.-W.)

**Keywords:** pediatric acute lymphoblastic leukemia, heme oxygenase-1, chemotherapy induced neutropenia, minimal residual disease

## Abstract

Whilst the survival rates of childhood acute lymphoblastic leukemia (ALL) have increased remarkably over the last decades, the therapy resistance and toxicity are still the major causes of treatment failure. It was shown that overexpression of heme oxygenase-1 (HO-1) promotes proliferation and chemoresistance of cancer cells. In humans, the HO-1 gene (*HMOX1*) expression is modulated by two polymorphisms in the promoter region: (GT)n-length polymorphism and single-nucleotide polymorphism (SNP) A(−413)T, with short GT repeat sequences and 413-A variants linked to an increased HO-1 inducibility. We found that the short alleles are significantly more frequent in ALL patients in comparison to the control group, and that their presence may be associated with a higher risk of treatment failure, reflecting the role of HO-1 in chemoresistance. We also observed that the presence of short alleles may predispose to develop chemotherapy-induced neutropenia. In case of SNP, the 413-T variant co-segregated with short or long alleles, while 413-A almost selectively co-segregated with long alleles, hence it is not possible to determine if SNPs are actually of phenotypic significance. Our results suggest that HO-1 can be a potential target to overcome the treatment failure in ALL patients.

## 1. Introduction

Acute lymphoblastic leukemia (ALL) is one of the most common hematological neoplasms that affects the entire lifespan of both infants and adults, with significant prevalence between the ages of 2 and 6 years. Among children, more than 80% cases of ALL are B-cell precursor ALL (BCP-ALL) [1]. Prognosis and outcome in ALL depend on several factors including: age, genetic aberrations and gene expression profile, prednisone response, leukemia characteristics and last but not least—an initial leukemic cells responsiveness to the induction therapy, defined as minimal residual disease (MRD), that is the strongest independent risk factor for relapse in childhood ALL [1,2,3]. Since MRD is a measurement of drug resistance in vivo and reflects multiple cellular, host, and treatment variables, it has been therefore used for refining initial treatment stratification to the risk categories (standard (SR), intermediate (IR), and high risk (HR)), allowing to tailor the intensity of chemotherapy for the individual patient [4]. Although survival rates of childhood BCP-ALL have increased remarkably, reaching up to 90% in developed countries [5,6], therapy resistance and toxicity are still the most important barriers to survival, associated with high morbidity and mortality [7,8,9,10]. Among them, neutropenia (chemotherapy-induced neutropenia—CIN) [11] results in a high risk of life-threatening infections and therefore is the primary cause of dose reductions and dose delays during chemotherapy [12]. For the treatment and prophylaxis of CIN, human recombinant G-CSF (granulocyte colony stimulating factor) is recommended [13,14]. To guide more efficient and cost-effective applications of the G-CSF, a number of studies have attempted to define the precise risk factors for CIN [15]. Although the frequency of CIN is related to some non-genetic variables, including chemotherapy scheme, cancer type and such patient-specific factors as age, presence of concomitant diseases and general health status [16,17], little is known about the genetic factors affecting susceptibility to chemotherapy-induced myelosuppression [18,19].

Some chemotherapeutic agents that might be used for ALL treatment (such as doxorubicin, vinblastine or camptothecin) [20] increase the level of oxidative stress, as their antitumor activity depends directly on H_2_O_2_-induced apoptosis [21,22,23,24,25]. One of the critical cytoprotective proteins induced in response to oxidative stress is heme oxygenase-1 (HO-1, encoded by *HMOX1* gene localized on chromosome 22q12) [26,27,28,29,30]. HO-1 is a heme-degrading enzyme yielding ferrous iron, biliverdin and carbon monoxide. It exerts antioxidative and antiapoptotic effects in response to various cellular stressors [31]. In carcinogenesis, HO-1 has an ambiguous role [32,33,34]. Even though it is essential for preventing carcinogenesis in healthy cells through maintaining redox homeostasis [35], under the ongoing process of tumorigenesis, its activation becomes deleterious for patients, since its antioxidant, antiapoptotic, and anti-inflammatory properties promote proliferation and invasiveness of malignant cells, leading ultimately to protection of neoplastic cells from apoptosis [36], which was also observed in AML [29]. Furthermore, HO-1 regulates proliferation and differentiation of many cell types [37,38,39,40,41,42,43,44], and recently we have demonstrated that it also affects granulocytic development through influencing myelocyte proliferation [45], suggesting its potential role in regulating granulopoiesis under stress-induced conditions, such as chemotherapy.

In human, the level of inducible HO-1 response is controlled by polymorphisms in the gene promoter region [46]. Based on the (GT)n microsatellite length polymorphism, the *HMOX1* promoter can be classified into three categories: short, medium and long [47]) and based on the various promoter lengths, six genotypes are derived: short/short (SS), medium/short (MS), medium/medium (MM), long/short (LS), long/medium (LM), and long/long (LL) [48], affecting both basal and induced levels of HO-1 expression [49]. Since the long (GT)n sequence has the potential to acquire thermodynamically unfavorable Z-DNA conformation [46], therefore, short alleles were shown to be associated with increased transcriptional activity, higher inducibility and enhanced HO-1 enzymatic activity in numerous biological and clinical studies [46,47,49,50,51,52,53,54,55]. Interestingly, it was also demonstrated in lymphoblastoid cell lines that the cells with short (GT)n repeats exhibited an increased resistance to oxidant-induced apoptosis [51]. The association between *HMOX1* promoter polymorphisms and cancer susceptibility has been studied widely, but remains inconsistent [56]. Both LL genotype [52,54,57,58,59,60] and SS genotype [61] were shown to increase risk of different cancers. The second type of polymorphism in *HMOX1* gene is associated with an A(−413)T (rs2071746) single-base change [47,62]. In this case of SNP A(−413)T, it was suggested that the A might be associated with a higher promoter activity than the T allele [46,63], but the contradictory results were also published [64]. There are only a few studies investigating both types of polymorphisms (lengths and SNPs) simultaneously [65] and there are no data on the effect of certain polymorphic variants on the clinical course in cancer patients, including ALL patients.

The aim of this study was to investigate the impact of *HMOX1* promoter polymorphisms on long-term clinical outcomes, the prevalence of childhood ALL, and the role of certain polymorphic variants in resistance to chemotherapy. Second, since HO-1 plays a role in granulopoiesis, and we have verified whether those polymorphisms may reflect a predisposition to CIN incidences in children with ALL.

## 2. Results

### 2.1. Length and A(−413)T SNP Polymorphisms and Risk of Relapse

**Figure 1:**
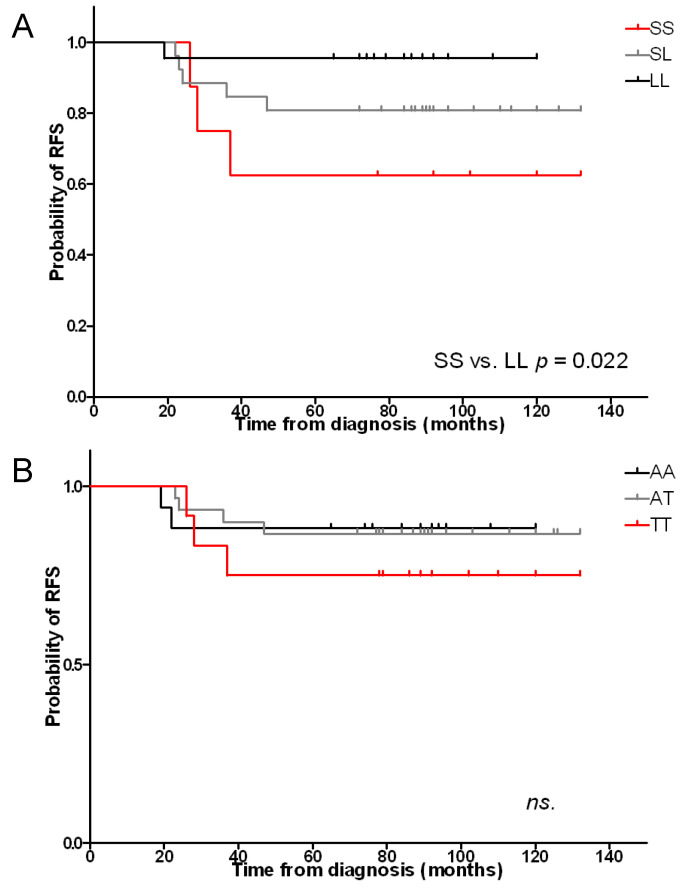
Relapse-free survival by grouping patients according to the presence of (**A**) length polymorphism and (**B**) SNPs polymorphisms. (**A**) The 5-year actuarial probability of relapse-free survival was 62.5%, 80.7% and 95.5% for patients with SS (red line, *n* = 8), SL (gray line, *n* = 26) and LL (black line, *n* = 22) genotype, respectively. (**B**) The 5-year actuarial probability of relapse-free survival was 88.2%, 86.7% and 75% for patients with TT (red line, *n* = 12) genotype, AT (gray line, *n* = 30) and AA (black line, *n* = 17), respectively.

To investigate the long-term clinical impact of certain polymorphisms, we evaluated the trend of standardized log-rank statistics using relapse-free survival (RFS) time. Among patients with the SS genotype, 3 patients (37.5%) experienced relapse at mean time of 30 months from the diagnosis. Among patients with the SL genotype, five patients (18.5%) experienced relapse at mean time of 30.4 months from the diagnosis. Among patients with the LL genotype, one patient (4.8%) experienced relapse at 19 months from the diagnosis. The proportion of patients who experienced relapse within certain subgroup (SS, SL or LL) did not reach statistical significance (*p* = 0.08). The five-year actuarial probabilities of RFS were 62.5%, 80.8%, and 95.5% for SS, SL, and LL group respectively, with the highest difference between SS vs. LL genotype (*p* = 0.022) (Figure 1A). This analysis suggests that the presence of short alleles might be a risk factor for poor prognosis. However, the effect of length polymorphism on patients’ overall survival (OS) did not reach statistical significance.

Among patients with the AA genotype, two patients (11.8%) experienced relapse at mean time of 20.5 months from the diagnosis. Among patients with the AT genotype, four patients (13.3%) experienced relapse at mean time of 32.5 months from the diagnosis. Among patients with the TT genotype, three patients (25%) experienced relapse at mean time of 30.3 months from the diagnosis. The proportion of patients who experienced relapse within certain subgroup (AA, AT or TT) was statistically insignificant. The five-year actuarial probabilities of RFS were 88.2%, 86.7%, and 75% in AA, AT, and TT group respectively, not showing statistical difference (Figure 1B). This analysis indicates that A(−413)T polymorphism has no prognostic significance.

### 2.2. Co-Segregation of A(−413)T SNP with Length Polymorphisms

**Figure 2:**
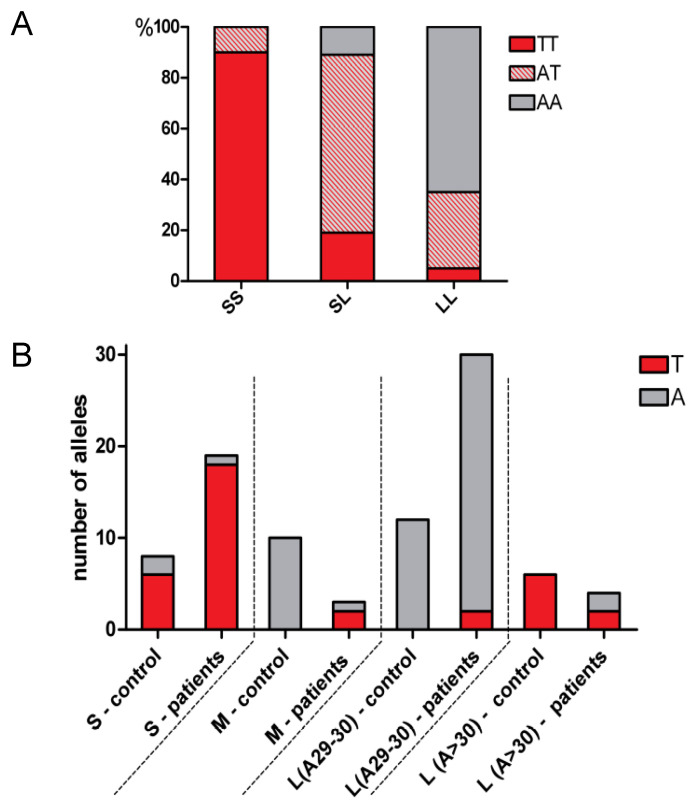
Co-segregation of the A and T alleles with *HMOX1* promoter length variants: (**A**) Shown as a percentage frequency distribution of AA, AT and TT genotype in SS, SL and LL individuals (both patients and control group); (**B**) distribution of AA and TT in individuals with certain length variants, separately in the patient and control group; S—short alleles (GT *n* < 24), M—medium alleles (24–28 GT *n*), L—long alleles (29/30 GT *n*), L—very long alleles (GT *n* > 30).

In order to examine the co-segregation of the *HMOX1* −413 A > T SNP with (GT)n repeat polymorphisms we compared a percentage frequency distribution (frequency of AA, AT or TT genotype distribution, shown as a percentage of the total frequencies in all analyzed subjects divided on the basis of length polymorphism (Figure 2A)) and distribution of different (GT)n alleles in subjects homozygous in terms of TT or AA (Figure 2B). Concordantly with data obtained by Bean et al. [65], we found that both in healthy controls and in ALL patients, in almost all individuals short alleles occurred simultaneously with T alleles, and in most cases long alleles (29/30 GT) were present together with A alleles (Figure 2A). We also noticed that in the control group, very long alleles (>30 GT) exclusively occurred together with T alleles (Figure 2B). Because of this co-segregation, without simultaneous investigation of SNP and length polymorphisms, it is not possible to clearly identify which of the A or T alleles exhibit transcriptional activation properties. Due to this doubt, and also the fact that the length polymorphism function has been well documented, and that our data demonstrate its role in RFS, we postulate that only the length polymorphism, but not SNP, should be considered clinically significant.

### 2.3. Distribution of the Length Polymorphisms and Acute Lymphoblastic Leukemia Prevalence

**Figure 3:**
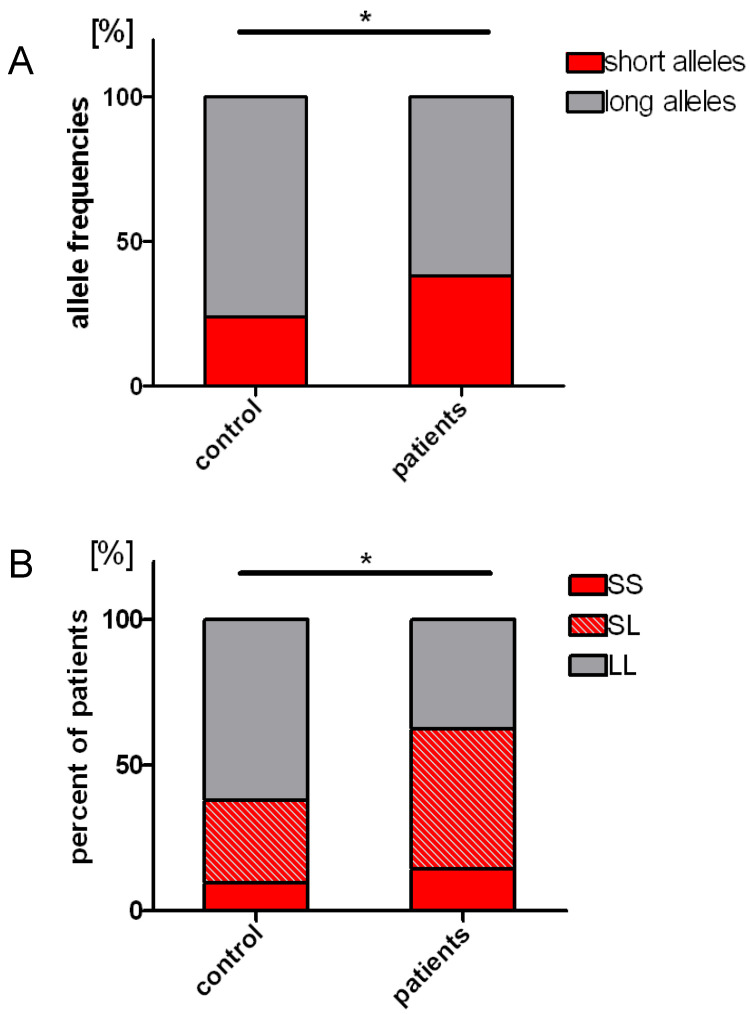
(**A**) Frequencies of short and long alleles in the patient and control groups. In ALL patients: 43 short alleles (38.4%) and 69 long alleles (61.6%); in control group: 20 short alleles (23.8%) and 64 long alleles (76.2%). (**B**) Comparison of percentage of patients and healthy controls with the SS, SL or LL genotype. In ALL patients: SS genotype—8 patients (14.3%), SL genotype—27 patients (48.2%), LL genotype—21 patients (37.5%). In controls group: SS genotype—4 individuals (9.5%), SL genotype—12 individuals (28.6%), LL genotype—26 individuals (61.9%). *—*p* < 0.05.

Because the criteria for distinguishing the M and L alleles are inconsistent (different cutoffs set arbitrary in different studies), and their precise distinction is not of biological and clinical importance, we analyzed our results applying only 2 allele categories (S and L), with a cutoff for S alleles of 23 or fewer dinucleotide repeats (<24), based on our earlier research where we experimentally determined the cut-off value in human endothelial cells, by measuring *HMOX1* expression [49]. Next, we assessed the frequency of the S and L alleles in control subjects and in patients (Figure 3A). We found that L alleles were prevailing over S alleles in both control and ALL patients’ groups. However, S alleles were statistically more frequent in ALL patients than in healthy children (38.4% in ALL patients and 23.8% in control group, *p* = 0.032 Figure 3A).

Then we compared the proportion of individuals with the SS, SL and LL genotype in the studied groups. Since it had previously been shown that one short allele would suffice to provide higher activation of *HMOX1* promoter [49,66], in the first step we combined individuals with the SS and SL genotypes into one group. We found that among ALL patients, the proportion of such individuals was statistically higher than in the control group of healthy people (62.5% in ALL patients; 38.1% in control group, *p* = 0.024 Figure 3B). Because in lymphoblastoid cell lines (LCLs) it was shown that *HMOX1* mRNA expressions and HO-1 activities were significantly higher in cells with the SS genotype [67], in the next step we compared a proportion of homozygous SS individuals in studied groups. However, we did not find significant difference in the distribution of SS homozygotic individuals between ALL and control group (14.3% in ALL patients; 9.5% in control group, Figure 3B). These results indicate that the presence of the S allele (SS or SL genotype) may increase the risk of ALL leukogenesis, but the additional presence of a second S allele (SS genotype) is no longer relevant.

### 2.4. Length Polymorphisms and Treatment Response

**Figure 4:**
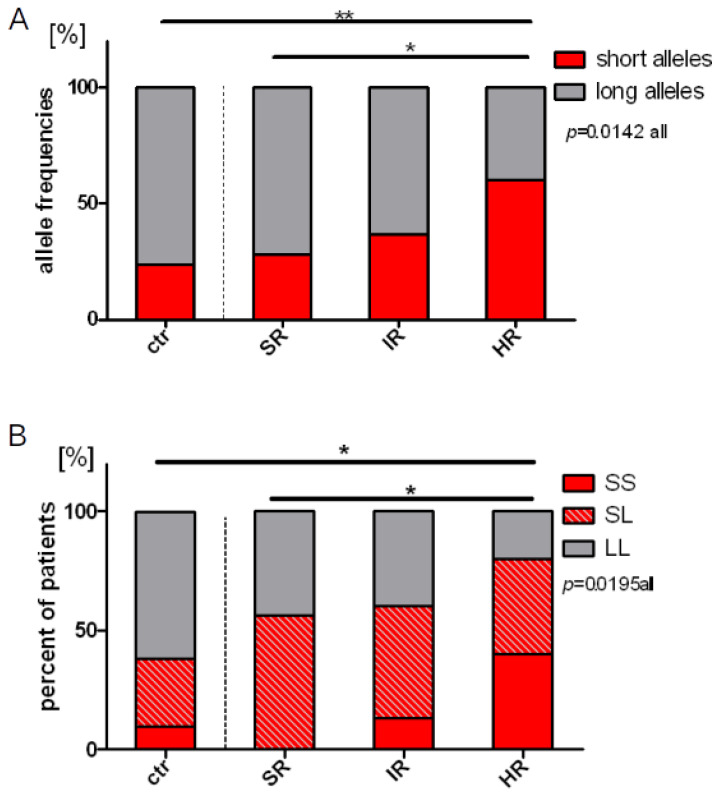
(**A**) Frequencies of short and long alleles in the control group and in patients stratified into the risk groups. In the control group: 20 short alleles (23.8%) and 64 long alleles (76.2%); in the SR group: 9 short alleles (28.1%) and 23 long alleles (71.9%); in the IR group: 22 short alleles (36.7%) and 38 long alleles (63.3%); in the HR group: 12 short alleles (60%) and 8 long alleles (40%); (**B**) Comparison of percentage of individuals with the SS, SL and LL genotypes in the control group and in patients stratified into the particular risk groups. In the control group: SS genotype—4 individuals (9.5%), SL genotype—12 individuals (28.6%), LL genotype—26 individuals (61.9%). In the SR group: SS genotype—0 patients, SL genotype—9 patients (56.2%), LL genotype—7 patients (43.8%). In the IR group: SS genotype—4 patients (13.3%), SL genotype—14 patients (46.7%), LL genotype—12 patients (40%). In the HR group: SS genotype—4 patients (40%), SL genotype—4 patients (40%), LL genotype—2 patients (20%). *—*p* < 0.05; **—*p* < 0.01.

Next, we found different frequency of the S and L alleles in the risk-adjusted patient groups (*p* = 0.014). The frequency of the S alleles was increased concomitantly with rising risk stratification in the groups, with a statistically higher frequency of the S alleles in HR patients when compared to the control group (*p* = 0.003) or to the SR group (*p* = 0.041) (23.8% in the control group; 28.1% in the SR group; 36.7% in the IR group; 60% in the HR group, Figure 4A). Next, we compared the proportion of the SS, SL, and LL genotypes in the patients versus the control group, and found an unequal distribution of those genotypes (*p* = 0.014). When we combine individuals with the SS and SL genotypes (as individuals with at least one short allele) and compared their proportion between the risk groups, we found the highest number of such patients in the HR group, with significant difference when compared to the control group (*p* = 0.031) (38.1% in the control group; 56.2% in the SR group; 60% in the IR group; 80% in the HR, Figure 4B). Next, we compared the proportion of homozygous SS individuals in all studied groups. The highest proportion of homozygous SS genotype was observed in the HR risk group, with significant difference when compared to healthy control (*p* = 0.035) and to the SR group (*p* = 0.014) (9.5% in the control group; 0% the SR group; 13.3% in the IR group; 40% in the HR group, Figure 4B). These results indicate that the presence of short alleles, especially in the homozygous configuration, is associated with stratification to the HR group, reflecting a higher risk of treatment failure, coherent with a higher risk of relapse (Figure 1A).

**Figure 5:**
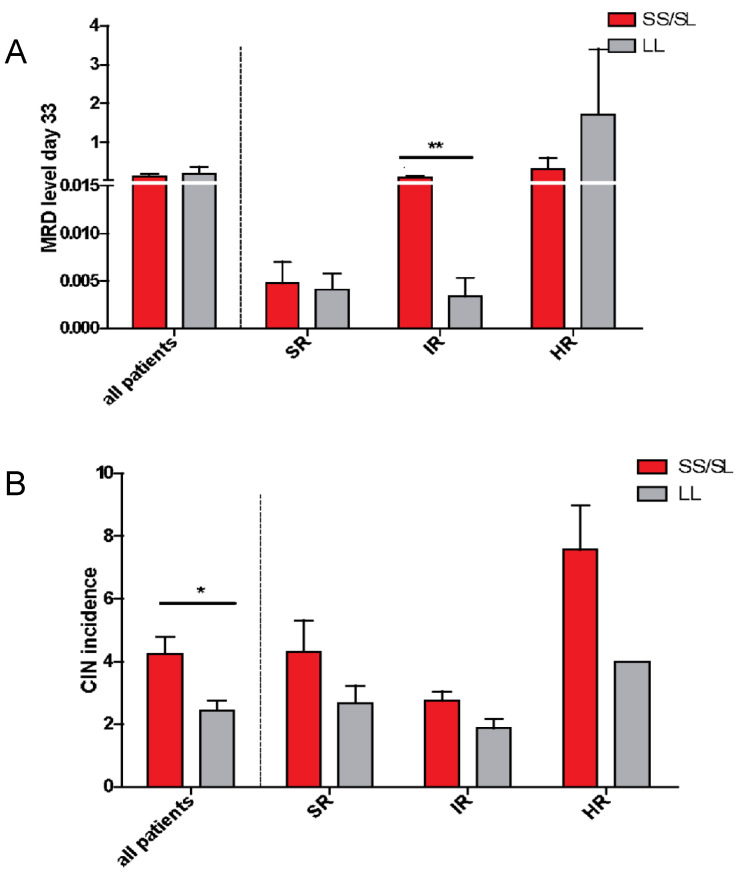
(**A**) MRD level at 33rd day of treatment in association with the presence of short alleles in patients, shown in all patients and separately in patients stratified into the particular risk groups. Mean MRD was 0.086 ± 0.17 in patients with at least one S allele versus mean MRD 0.0033 ± 0.006 in the LL patients. In the SR group—data available for 15 patients, in the IR group for 28 patients, in the HR group for 10 patients. (**B**) CIN incidences in association with the presence of short alleles in patients, shown in all patients, and separately in patients stratified into the particular risk groups. Then the influence of genotype and a risk group was calculated as independent variables on a number of CIN incidents. It appeared that the main effect resulted from the risk group (two-way ANOVA, *p* = 0.0005), but there was also an effect of genotype (*p* = 0.0055). In the SR group—data available for 15 patients, in the IR group for 24 patients, in the HR group for 10 patients. Data are presented as means and SEM * p < 0.05, ** *p <* 0.01.

We also investigated whether the presence of the S allele could indeed lead to a higher blast cells persistence, measured as MRD. On the 15th day of treatment there was no statistical difference in blast count between patients characterized by the presence of at least one S allele (data not shown). However, on the 33rd day, in the IR group we found that SS/SL patients exhibited higher MRD values (*p* = 0.009, Figure 5A). Nevertheless, as the difference was found only in the IR group, and only at day 33rd, we doubt that these data might be biologically relevant.

### 2.5. Chemotherapy-Induced Neutropenia (CIN)

Subsequently, we checked whether the frequency of G-CSF administration cycles, which reflects the number of CIN incidents in a patient, was related to the presence of the certain *HMOX1* promoter allele. In the first step, the analysis was carried out in all patients, regardless of the risk group. It was found that patients with the S allele had significantly more neutropenic events that had to be treated by administering G-CSF (*p* = 0.048, Figure 5B). However, in the HR group, most G-CSF administrations are pre-scheduled in advance, and in the SR and IR groups G-CSF is administrated if necessary. Because the genotype itself relates to the risk groups, we examined the influence of genotype and risk group on a number of CIN incidents as independent variables. We found that the main effect resulted from the risk group (two-way ANOVA, *p* < 0.001), but we also found the effect of genotype (*p* = 0.006). Since each risk group is treated with a different therapeutic protocol, and a given type of chemotherapeutic regimens may per se predispose to a different occurrence of CIN, the analysis was also conducted separately in the risk subgroups. Accordingly, in all risk groups the number of cycles of G-CSF treatment tended to be lower among children with the LL genotype, however it was not statistically significant (Figure 5B).

### 2.6. HO-1 Expression and Its Localization within Normal Human Hematopoietic System

In the last set of experiments, we wanted to assess a possible biological mechanism responsible for a distinct clinical course in patients with different length polymorphisms of *HMOX1* promoter. We wanted to assess whether it might be associated with the role of HO-1 in the blast cells *per se*, or rather in the myeloid compartment.

Since the physiologically relevant HO-1 suppressor—heme-regulated BACH2—is a critical negative mediator at the pre-B cell receptor checkpoint and is a safeguard against leukemogenesis, we addressed the question whether HO-1 might also play a role in physiological lymphopoiesis. For this purpose, we examined the HO-1 expression and its localization within normal human hematopoietic system. We sorted cells of specific immunophenotype and stained HO-1 protein to check its expression by means of confocal microscopy. To distinguish human hematopoietic progenitor subsets, the staining scheme described by Seita et al. was employed [68]:

1. Hematopoietic stem cells (HSC): Lin^−^CD34^+^CD38^−^CD90^+^CD45RA^−^
2. Multipotent progenitors (MPP): Lin^−^CD34^+^CD38^−^CD90^−^CD45RA^−^
3. Common lymphoid progenitors (CLP): Lin^−^CD34^+^CD38^−^CD10^+^
4. Common myeloid progenitors (CMP): Lin^−^CD34^+^CD38^+^CD123^+/low^CD45RA^−^
5. Megakaryocyte-erythroid progenitor (MEP): Lin^−^CD34^+^CD38^+^CD123^−^CD45RA^−^
6. Granulocyte-macrophage progenitor (GMP): Lin^−^CD34^+^CD38^−^CD123^+^CD45RA^+^
7. Lymphoid maturation steps were established based on:
  - preBI: CD19^+^CD34^+^CD10^+^CD20^−^
  - preBII: CD19^+^CD34^−^CD10^+^CD20^dim^
  - Immature B cells: CD19^+^CD34^−^CD10^+^CD20^+^
  - Mature B cells: CD19^+^CD34^−^CD10^−^CD20^+^

**Figure 6:**
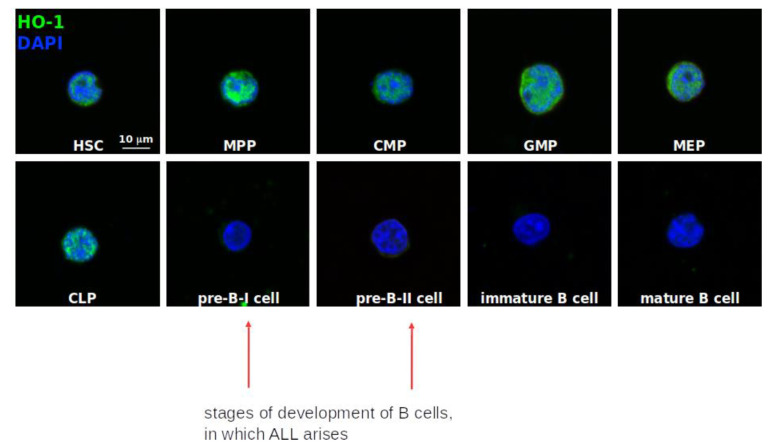
Confocal microscopy slides displaying HO-1 protein (green) and nuclear staining (blue) in normal bone marrow hematopoietic stem and myeloid progenitor cells, as well as B-cell developmental stages. HSC—hematopoietic stem cells, MPP—multipotent progenitors, CMP—common myeloid progenitors, GMP—granulocyte-macrophage progenitor, MEP—megakaryocyte-erythroid progenitor, CLP—common lymphoid progenitor.

Interestingly, we found that in hematopoietic stem cells and in progenitors committed to granulocyte or megakaryocyte lineage HO-1 is mainly located in the nucleus, what may indicate its non-canonical, non-enzymatic role [69]. However, upon maturation into preB-I stage of B-cell development (the stage of normal B-cell development, which is most prone to malignant transformation into BCP-ALL (the pre-pro-B to pre-BI transition) [70], HO-1 expression was barely detectable (Figure 6). This was consistent with the fact that BACH2—HO-1 suppressor—is upregulated at the transition from multipotent progenitor (MPP) into the B cell lineage, before the completion of the heavy chain checkpoint [71]. These data suggest that in physiological conditions HO-1 expression is not required for the maturation of B-cell precursors.

### 2.7. HO-1 Expression in Peripheral Blood and Bone Marrow Cells before and during Chemotherapy

**Figure 7:**
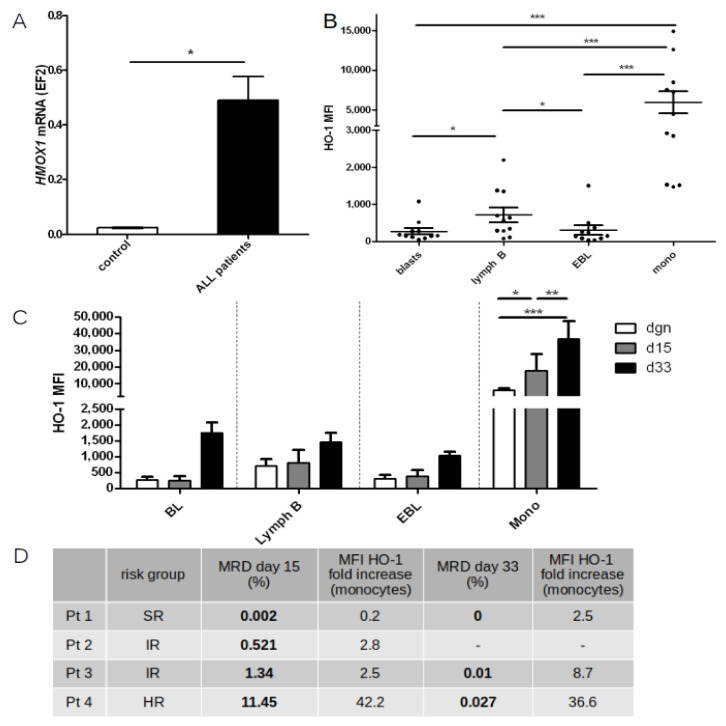
(**A**) Comparison of *HMOX1* mRNA level in peripheral blood samples taken from healthy, untreated children (*n* = 3) and from ALL children (*n* = 19) during treatment course, at stage of clinical remission when no blast cells were found in the blood, and complete blood count was normalized. (**B**) Basal HO-1 expression (shown as MFI) in leukemic cells and the residual normal cell subsets present in the sample at the day of diagnosis (*n* = 11). (**C**) MFI of HO-1 in each cell subset before treatment (*n* = 11) and at day 15th (*n* = 6) and 33rd (*n* = 3) of induction. (**D**) The fold increase of HO-1 expression in monocytes in relation to the risk group and to treatment response, expressed as MRD level. Blasts—leukemic cells, lymph B—mature lymphocytes, EBL—erythroblasts, mono—monocytes. *—*p*< 0.05; **—*p* < 0.01; ***—*p* < 0.001.

To check whether chemotherapy protocols used in ALL patients actually induce HO-1 expression in normal cells, regardless of the presence of leukemic cells, *HMOX1* mRNA level was measured in peripheral blood samples taken from ALL patients during treatment course, but at the stage of clinical remission, when no blast cells were found in the peripheral blood, and complete blood count was normalized. We found that in comparison to healthy, untreated children, in ALL patients the level of *HMOX1* mRNA was significantly elevated (*p* = 0.017, Figure 7A). It must be noted, however, that *HMOX1* mRNA measurements were performed in samples taken from patients at various stages of treatment (including patients just before the end of treatment or after the end of entire treatment course). Hence, in this case many additional factors might have influenced the elevation of *HMOX1* expression, not solely the chemotherapeutic agents.

In the next step we compared the level of HO-1 expression (presented as mean fluorescence intensity—MFI) in certain cell subsets within bone marrow at the day of diagnosis (prior to treatment—day 0) and on days 15 and 33 of treatment. To exclude cellular debris, only nucleated cells (syto41^+^) were taken into analysis. Leukemic blasts were identified as immature B cell population with aberrant immunophenotype, defined individually for each patient (leukemia associated immunophenotype—LAIP). Normal mature B cells were defined as cells within “lymphgate” (bright CD45 expression, low SSC) positive for CD19 and CD20 but negative for CD10 and CD34. Erythroblasts were defined as cells negative for CD45 with low SSC characteristics, simultaneously negative for CD11a, CD19, CD10, CD34, and CD20. Monocytes were defined as cells within “monogate” (bright expression of CD45 and intermediate SSC), negative for CD19, CD10, CD20, positive for CD38, with very bright CD11a expression. Each population was backgated on FSC/SSC dotplot.

At the day of diagnosis we compared the HO-1 MFI between leukemic cells and the residual normal cell subsets present in the sample (Figure 7B). We found that HO-1 expression was the lowest in leukemic blasts, with comparable level of expression as in erythroblasts. The expression of HO-1 in mature B-cells was slightly higher than in the blast cells (*p* = 0.004), and erythroblasts (*p* = 0.048). The highest HO-1 expression was detected in monocytes when compared to all analyzed subsets (monocytes in comparison: to blast cells—*p* < 0.0001, to mature lymphocytes B—*p* = 0.0002, and to erythroblasts—*p* = 0.0001). This pattern of HO-1 expression in non-malignant cells was similar to pattern of *Hmox1* mRNA expression described in murine hematopoiesis model [72]. As HO-1 is a stress inducible enzyme, we have investigated whether HO-1 expression is indeed elevated in patients during induction chemotherapy, as at this point the regimen intensity is the same, regardless of the risk group. This analysis was done in six patients at day 15th and only three patients at day 33rd, as in the remaining three patients there was an insufficient amount of sample (due to aplastic bone marrow) to perform additional stainings, apart from routine MRD measurement. Only in monocytes was the HO-1 level statistically higher during following induction cycles (two-way Anova with Bonferroni posttests, Figure 7C), we performed further analysis tracking relative changes of HO-1 level in individual patients with distinct early treatment response (Figure 7D). We found that the higher the fold increase of HO-1 in monocytes was observed, the higher level of MRD detected. Based on both HO-1 level directly measured in monocytes at days 15th and 33rd as well as relative HO-1 level changes in individual patients with different early treatment response (expressed as MRD level), we postulate that the altered HO-1 expression in bone marrow monocytes—but not in lymphocytes—may represent a response to stressful conditions, rather reflecting modulation of HO-1 in the tumor microenvironment as a potential mechanism of chemotherapy resistance.

### 2.8. Supplementary Information—A-SNP-T Polymorphism

Because cut-off point criteria of short, medium and long alleles are inconsistent, we also demonstrated our data as raw values (Supplementary Figure S1). The investigation of A(−413)T SNP (Supplementary Figure S2) revealed no significant difference in the T and A alleles frequencies in ALL patients compared to the control group (Supplementary Figure S3A). We also compared the proportion of the AA, AT and TT genotypes in patients and controls and we did not find statistically significant difference (Supplementary Figure S3B). Similarly, the frequency of alleles A and T between the risk groups was not altered significantly, as well as the proportion of patients with certain genotype AA, AT or TT (Supplementary Figure S4A,B). In the HR group there were only slightly more patients displaying at least one T allele or displaying homozygous TT genotype. However, this result can be attributed to the effect of co-segregation of the T allele with short alleles (which were predominant in the HR group), than to regulatory properties of the T allele per se. Since in contrast to unequivocal S allele distribution, the final distribution of T allele in patients was balanced by the presence of T alleles co-segregated with very long alleles in control group, the difference between the groups was lost.

## 3. Discussion

Given the concerns about the two most important barriers for survival of BCP-ALL children—therapy resistance and treatment-induced toxicities—the research on predictive markers is of great interest to further guide clinicians on dose individualization during personalized therapy. Recently, some studies explored the prognostic significance of HO-1 expression in human cancers, as well as its possible correlation with tumor clinical features and outcome [73]. The HO-1 expression depends on common *HMOX1* promoter polymorphisms [74,75]. Because such a universal modulatory mechanism seems to be an accurate, objective, easy to perform, and a convenient routine clinical utility, we decided to verify the role of *HMOX1* promoter polymorphisms as prognostic marker candidates in BCP-ALL children. It has been confirmed in several studies (also from our group: [49]) that HO-1 expression depends on the length of microsatellite sequences in *HMOX1* promoter. The lower number of GT repeats allows for a higher HO-1 expression under control conditions and a stronger induction in response to stress. [46,47,50]. Several clinical studies have demonstrated that the length of GT region is associated with increased susceptibility to some human diseases [46]. In contrast, only sparse research has investigated an A(−413)T SNP in terms of clinical investigations [76] and its impact on *HMOX1* transcription. To exacerbate, they provide contradictory results, indicating that either A [63] or T [64] is responsible for a higher transcriptional activation. Therefore, in this study we performed both genotyping of the (GT)n microsatellite polymorphism and A(−413)T SNP in the *HMOX1* gene promoter in patients with BCP-ALL and in control group of healthy children.

In accordance with the previous data [65], we found high co-segregation of specific subtype of (GT)n microsatellite polymorphisms with A(−413)T SNP variants, both in the control group and in ALL patients. Subjects carrying the most common subtype of L allele (29–30(GT) repeats) possessed mainly A allele, whereas individuals with short (<24 GT) or very long (>30 GT) alleles exhibited very high prevalence of T variant of SNP. 100% co-segregation of the homozygotes SS (considered as more transcriptionally active) with homozygous TT (regarded as less transcriptionally active), and the co-segregation of the most common type of L allele (shown to have low transcriptional activity) with the A allele, might lead to a conclusion that both types of polymorphisms occur usually in a configuration that abolishes their mutual effects. Importantly, most of the research on the clinical significance of SNP polymorphism has not simultaneously investigated the length polymorphisms, so it cannot be ruled out that the described differences, attributed to the certain SNP, could have in fact resulted from co-segregation of SNP with the specific subtypes of length promoter allelic variants which has a predominant role. Only the comparison of homozygous SS patients and homozygous AA or TT patients would fully answer the question. However, the coexistence of the SS alleles with the AA alleles is extremely rare [65], and in our study group there were no patients with such genotype. Due to this inconsistency and difficulties in SNP polymorphism interpretation, we postulate that only the GT microsatellite polymorphism can be considered clinically significant. Analyses carried out as a part of this work also proved the lack of clinical relevance of A(−413)T SNP variants, as there were no differences in RFS rate and A(−413)T SNP distribution among the studied groups.

In its important to note that the literature does not propose consistent classification of the (GT)n length polymorphism [48]—while some studies comprise to “short” category alleles shorter then 25, it was confirmed in molecular studies that promoter with less than 24 (GT) repeats shows both increased HO-1 basal promoter activity and elevated response to stimuli [46,47,49,50]. Hence, in this work, we also used the cut-off <24 GT as “short” category. However, after recalculation of data using cut-off point less than 25 GT, we also obtained a very similar distribution of results, since there were very few patients with the 24 GT allele (data not shown). In term of category “medium” and “long” the exact cut-off point varies even more between studies [46,47,50]. However, precise discrimination between these alleles does not bring any additional information, as it was confirmed that biologically important modulation of HO-1 activity depends on the presence of the short allele [49,66]. Hence, in this study, we also focused on the classification of *HMOX1* promoter based on short and long alleles.

First, we examined the association of polymorphic variants of the *HMOX1* promoter with RFS. We found that patients with the SS genotype display the poorest RFS, especially when compared with the LL genotype. The SL genotype displayed intermediate risk of relapse. We also analyzed the length polymorphisms in association with childhood ALL prevalence. We demonstrated that in comparison to the control group, in BCP-ALL patients both the S (GT)n allele frequency and the proportion of patients with at least one S allele (SS genotype combined with SL as one group) were higher. However, there was no difference in the proportion of patients and controls with the SS genotype. It indicates that short, more active variants of the *HMOX1* promoter, not only do not protect against the initiation of leukogenesis, but on the contrary, may even promote carcinogenesis. Studies on the association between the *HMOX1* promoter polymorphism and the prevalence of different types of cancer in humans are inconsistent, showing that the same allelic variants seem to be protective against one type of cancer, while posing high risk for other types [52,54,56,57,58,59,60,61,77,78,79]. Some discrepancies in the *HMOX1* promoter polymorphisms and cancer prevalence may stem from a complex physiological role of HO-1, variability of its expression in different tissues (http://www.proteinatlas.org/ENSG00000100292-HMOX1/tissue), and cell-type specific effects of HO-1 on cell differentiation [44,80]. This is especially true for such a complex and heterogenous tissues as bone marrow, where HO-1 is differently expressed in hematopoietic stem and progenitor cells and niche residing cells [72,81].

Although HO-1 is known to be critical for proper antibody production [82] little is known about its function in early B-cells development. Interestingly, its suppressor—BACH2 which is up-regulated at the transition from multipotent progenitor (MPP) to the pro-B [71]—has been shown to execute negative selection of premalignant early B cells that failed VH-DJH rearrangements at the pre-B cell receptor checkpoint. As it was previously suggested that loss of BACH2 in both normal pre-B cells and pre-B ALL may lead to leukemia [83], one could expect that HO-1 would be in turn elevated in leukemic cells. However, we found even decreased level of HO-1 in leukemic blasts in comparison to normal B cells and to monocytes. In normal bone marrow cells HO-1 was present from the stage of HSC, through MPP till CLP and after commitment to B lineage (at preB-I stage) HO-1 was barely detectable till the stage of mature B-cell (in contrast to the progenitors committed to granulocyte or megakaryocyte lineage). These findings were also consistent with the observation made in a murine model, showing that *Hmox1* mRNA level was extremely low at B-cell progenitor stage [72]. As HO-1 was almost undetectable at the stage which is most vulnerable to malignant transformation into BCP-ALL (the pre-pro-B to pre-BI transition [70]), and in the malignant cells its expression was very low, we doubt that HO-1 may play a significant role in protection of B progenitors from leukogenesis. Although it is highly unlikely that the presence of certain type of the promoter allelic variants affects HO-1 expression in B-cells and in leukemic blasts, it cannot be however, ruled out that HO-1 activity in stromal cells may indirectly affect B-cells e.g., through modulation of the level of free heme and iron availability in microenvironment [84,85].

HO-1 may be strongly induced in response to radiation, photodynamic therapy, or chemotherapy [33], pointing out the role of HO-1 in cancer chemoresistance [51]. As a better understanding of the mechanisms associated with resistance to therapy is essential to prevent tumor relapse, in the next step we checked the *HMOX1* (GT)n genotype distribution within BCP-ALL patients, depending on their relapse-risk group. We found that the frequency of the S alleles was higher is the HR group compared to the control group, as well as to the SR group. Subjects possessing at least one S allele of the *HMOX1* promoter were more frequently classified into the HR group than to the SR and IR groups. The *HMOX1* SS (GT)n homozygous genotype occurred mostly in the HR group, and has not been observed in the SR group. Additionally, leukemic blasts evaluation revealed that patients from the IR group, who carried at least single S allele, exhibited higher MRD at the 33rd day of therapy, which is consistent with the fact that drug resistance in vivo is reflected by the MRD level [1,2,3]. However, because in another risk group and at day 15th we did not observe a similar relationship, this observation may be biologically irrelevant.

Interestingly, HO-1 overexpression has recently been reported to negatively modulate glucocorticoid receptor pathways in prostate cancer cells [86], and glucocorticoids (prednisone and dexamethasone) play an essential role in the treatment of acute lymphoblastic leukemia (ALL) [87]. In our study, we did not find a significant difference in prednisone response between patients with certain *HMOX1* (GT)n genotype (data not shown). However, we showed that, in BCP-ALL children, chemotherapy upregulates HO-1 at both mRNA level (in total peripheral blood cells) and at protein level (selectively in monocytes and not lymphoid cells). Monocytes play an important role in mesenchymal stromal cell-driven immunomodulation. They differentiate into regulatory macrophages and produce many cytokines [88]. Therefore, we assume that HO-1 shifts in monocytes may also reflect macrophage polarization response and—to some extent—may represent shifts in stromal compartment. Hence, we speculate that unequal allelic variant distributions in BCP-ALL patients may be more important in stromal compartment. This supposition could be verified only by examination of trephine biopsy specimens, which are not taken routinely in ALL patients. Nevertheless, many studies have convincingly reported the role of HO-1 in tumor progression [74,89,90,91,92,93,94], pointing to its significance not only in tumor cells per se, but also in stromal compartment, particularly in the tumor-associated macrophages [95,96,97,98].

Interestingly, macrophages have also been shown to have a role in the mobilization of hematopoietic cells into the peripheral blood by G-CSF [99,100], and HO-1 induction has been reported to impair granulocyte mobilization [101]. Cytotoxic chemotherapy itself suppresses the hematopoietic system, dysregulating the physiological granulocyte proliferation and differentiation [102], and leading to chemotherapy-induced neutropenia (CIN) [11]. Hence, in the last step we investigated the effect of (GT)n microsatellite polymorphism on susceptibility to CIN incidents. We found that subjects with at least one short allele variant had significantly higher CIN incidents. Because different chemotherapeutic protocols are used depending on the risk group classification, we checked the length polymorphism distribution in BCP-ALL patients within different risk categories. However, only in the IR group did patients who carry at least single S allele reach a significantly elevated number of CIN incidents, most probably due to the fact that this group was the most numerous.

Under stress conditions, rapid enhancement of granulopoiesis is predominantly regulated by G-CSF-responsive transcription factor C/EBPβ [103]. C/EBPβ enhances proliferation [104], leading to fast adaptation of hematopoietic system to stress response [105]. A study by Suh et al. indicated that activity of C/EBP transcription factors can be inhibited by carbon monoxide (Suh, Jin et al. 2006), which was later confirmed by our group for C/EBPδ [44]. We have also shown a similar mechanism of indirect modulation of C/EBPβ in granulocytic development in HO-1 knock-out mice [45]. As HO-1 degrades heme with concurrent release of carbon monoxide, its increased expression governed by the short (GT)n variant of *HMOX1* promoter, might indirectly influence granulocyte maturation and myelocyte proliferation via C/EBPβ mediated pathway. In line with these results, we suppose that short allelic variant of (GT)n *HMOX1* promoter predisposes ALL patients to higher number of CIN incidents, and thus may help identify patients at greater risk for such complications. The suggested HO-1-dependent mechanism does not appear to be specific for certain drug, but is generally driven by oxidative stress, being one of the putative pathways that affect susceptibility to chemotherapy-induced myelosuppression.

Because genetic variations can be used to predict drug toxicity, safety, and efficacy [19,106], there have been attempts to identify patients at greater risk for CIN. Interestingly, genome-wide association studies (GWAS) performed in Japanese patients failed to identify genetic variants associated with CIN susceptibility that surpassed the genome-wide significance level [18]. However, only SNPs, but not microsatellite length polymorphisms, were investigated. Concordantly, in our study, we also did not find an association between SNP in the *HMOX1* promoter and CIN incidences (data not shown).

The limitation of our research is the relatively small number of patients. However, as our main aim was to check the influence of the *HMOX1* gene promoter polymorphisms on the treatment response and its toxicity, we selected a group of patients in whom the entire treatment protocol had been completed and the long follow-up data were available. Although this research brings some new data related to important therapeutic complications of ALL treatment, some of our results are of borderline statistical significance, hence they should be confirmed in larger study.

In summary, we described the effect of HO-1 and *HMOX1* gene promoter polymorphisms on the ALL development, chemotherapy resistance and patient risk to CIN incidents. We found that only (GT)n microsatellite polymorphism, but not A(−413)T SNP, is of clinical relevance. The presence of the short (GT)n allelic variant of the *HMOX1* promoter correlates with a higher risk and worse prognosis for ALL patients, and is associated with a higher rate of CIN incidents. Therefore, we propose that the presence of the short *HMOX1* alleles might help identify the high-risk patients. It also appears that the potential use of HO-1 inhibitors might be considered as a supplementary strategy in the treatment of ALL.

## 4. Materials and Methods

### 4.1. Patients

The study group comprised of 60 children with BCP-ALL treated in the Oncology and Hematology Department at the University Children’s Hospital of Krakow, between 2007 and 2015 with ALL IC-BFM 2002 or ALL IC-BFM 2009 therapeutic protocols. Patients were stratified into standard (SR, 18 patients, 30%), intermediate (IR, 32 patients, 53.3%) or high risk group (HR, 10 patients, 16.7%) based on the response to chemotherapy and disease characteristics (i.a. genetic factors, prednisone response). Children who had developed chemotherapy-induced neutropenia (CIN) were treated with G-CSF 24 h following incident until the neutrophil count achieved the level of at least 1000/µL. G-CSF regime ranged from 3 days to a maximum of one week. The frequency of CIN incidences in each patient was recorded. The control group comprised 42 children. Control group was selected from children referred to immunological outpatient clinic with upper respiratory tract infections, in whom after routine clinical and laboratory follow-up immunodeficiency was excluded. The study was approved by the Bioethics Committee of the Jagiellonian University (KBET/76/B/2014, 24 April 2014) and informed consent was obtained for all patients.

### 4.2. Patients’ Samples

Peripheral blood (PB) and bone marrow (BM) of patients were analyzed. BM samples were analyzed at 15th and 33rd days of treatment to assess the minimal residual disease (MRD) by multicolor flow cytometry. PB samples were taken by the end of treatment to assess *HMOX1* promoter polymorphisms and mRNA level.

### 4.3. DNA Isolation

DNA isolation was performed using Syngen Blood/Cell DNA Mini Kit according to the manufacturer protocol. Final DNA concentration was measured using NanoDrop spectrophotometric technique (Thermo Fisher Scientific, Wilmington, DE, USA).

### 4.4. Length HMOX1 Gene Promoter Polymorphism

In order to assess the number of (GT)n repeats in the *HMOX1* promoter region PCR reaction was done using GoTaq^®^G2 Flexi DNA Polymerase Kit (Promega, Madison, WI, USA) and the following pair of primers was applied: Forward: 5′-AGA GCC TGC AGC TTC TCA GA-3′, Reverse: 5′-ACA AAG TCT GGC CAT AGG AC-3′, where forward primer was labeled with 6-carboxyfluoresceine (FAM). PCR reaction was conducted under following conditions: 95 °C for 60 s, 37 cycles of 95 °C for 30 s, 58 °C for 15 s, 73 °C for 30 s and final elongation 73 °C for 45 s. DNA fragments analysis was performed by capillary electrophoresis (ABI PRISIM^®^ 310 Genetic Analyzer, Applied Biosystem, Foster City, CA, USA) with GeneScan^TM^ 350 ROX^TM^ dye Size Standard. Fragments sizes were determined with ABI Prism program (Gene Scan Analysis and Genotyper Software, Applied Biosystems, Foster City, CA, USA). In two patients from the SR group and in two patients from IR group, due to technical reasons it was impossible to determine length polymorphism, so data on length polymorphism are available for 56 children.

### 4.5. SNP HMOX1 Gene Promoter Polymorphism

For identification of SNP A(−413)T polymorphism PCR (GoTaq^®^G2 Flexi DNA Polymerase Kit (Promega, Madison, WI, USA)) was used with following primers and reaction conditions, Forward: 5′- GGA TGA ACC ATG AAA ATA CTA GAG TC-3′, Reverse: 5′-ATT TTG CTC CTT CCA GAG C-3′; 95 °C for 10 min, 34 cycles of 95 °C for 30 s, 56.1 °C for 30 s, 72 °C for 60 s and final elongation 72 °C for 10 min. To remove remaining primers and free nucleotides Exonuclease I treatment of PCR products was conducted using Exo-BAP Kit (Eurx, Gdańsk, Poland) for 15 min in 37 °C, followed by heat inactivation for 15 min in 80 °C. Sequencing PCR reaction was performed with BigDye^®^ Terminator v3.1 Cycle Sequencing Kit under conditions: 95 °C for 10 min, 25 cycles of 95 °C for 10 s, 50 °C for 5 s and 60 °C for 4 min. PCR products purification was carried out with BigDye^®^ X-Terminator Purification Kit (Applied Biosystems, Foster City, CA, USA). DNA fragments sequencing was analyzed by means of capillary electrophoresis AB3500 Genetic Analyzer (Life Technologies, HITACHI, Tokyo, Japan). In one patient from IR group, due to technical reasons, it was impossible to determine SNP polymorphism. Data on SNP polymorphism are available for 59 patients.

### 4.6. Cell Sorting, Immunofluorescent Staining and Confocal Analysis

In order to prepare immunofluorescence slides of certain hematopoietic stem or progenitor cell subsets, characterized elsewhere [68,107], normal BM samples were stained using following primary antibodies: Lin-FITC, CD90-PE, CD34-APC, CD38-AlexaFluor700, CD45-APC-H7, CD45RA-PE-Cy-7 or CD38-FITC, CD34-PE, CD19-APC, CD20-APC-H7 (BD Biosciences, San Jose, CA, USA). Cells of defined immunophenotype were sorted using MoFlo-XDP cell sorter (Beckman Coulter, Brea, CA, USA) into 20 mL PBS drops on poly-L-lysine coated slides. After settling, cells were fixed with 4% paraformaldehyde and permeabilized with 0.1% Triton X100. Afterwards, samples were incubated with 0.25% glycine for 30 min, followed by blocking with 3% BSA in PBS for 1 h. Subsequently, samples were stained overnight at 4 °C with primary antibodies recognizing HO-1 (rabbit polyclonal, SPA 896, Enzo, Warszawa, Poland) in a moisture chamber. After 5 washing steps, slides were incubated with goat anti-rabbit secondary antibody conjugated with Alexa Fluor 488 (Life technologies, Carlsbad, CA, USA) and DAPI for 1 h in darkness. After the next 5 washing steps, cells were analyzed using a Zeiss confocal microscope with ZEN Software (Zeiss, Oberkochen, Germany).

### 4.7. RNA Isolation, qRT-PCR

RNA isolation was performed using RNeasy^®^ Mini Kit. Reverse transcription and polyadenylation reaction were carried out with NCode™ VILO™ miRNA cDNA Synthesis Kit (Life Technologies, Carlsbad, CA, USA). Quantitative PCR reaction was conducted with StepOne Plus cycler (Applied Biosystems, Foster City, CA, USA) and SYBR Green JumpStart™ Taq ReadyMix™ (Sigma-Aldrich, St. Louis, MO, USA) under the following conditions: 95 °C for 10 min, 40 cycles of 95 °C for 30 s (denaturation), 60 °C for 60 s (starters annealing), 72 °C for 45 s (elongation) and 72 °C for 10 min (final elongation). The following primers were used: for *HMOX-1* Forward: 5′-TTC TTC ACC TTC CCC AAC ATT G-3′, Reverse: 5′-CAG CTC CTG CAA CTC CTC AAA-3′, and for EF-2 Forward 5′-GAG ATC CAG TGT CCA GAG CAG-3′, Reverse 5′-CTC GTT GAC GGG CAG ATA GG-3′ as an endogenous control.

### 4.8. MRD Detection Using Flow Cytometry

Multicolor flow cytometry analysis of BM was performed at the day of diagnosis to assess leukemic-associated immunophenotype (LAIP) of blasts cells. A following antibodies were used for cell immunophenotyping: CD34-PE, CD45-PerCP, CD10-PE-Cy7, CD19-APC, CD38-AlexaFluor-700, CD20-APC H7, CD11a-BV510 (BD Biosciences, Waltham, MA, USA), CD58 (Beckman Coulter, Brea, CA, USA). On the day 15th and 33rd BM was analyzed using the same panel of antibodies to determine MRD. To the appropriate amount of BM (10^6^ cells) antibodies listed above were added, samples were incubated for 20 min at room temperature in darkness. Erythrocytes were lysed for 10 min at room temperature in darkness with lysing solution (BD FACS Lysing Solution, Becton Dickinson Biosciences, San Jose, CA, USA), washed twice in PBS, and finally resuspended in 200 μL of PBS. For distinguishing nucleated cells, samples were stained with Syto^®^41 (Thermo Fisher Scientific, Waltham, MA, USA). FACS analysis was done using FACSCanto or FACSCanto10 with FACSDiva Sorfware v. 8.1 (Becton Dickinson Biosciences, San Jose, CA, USA).

### 4.9. HO-1 Detection Using Flow Cytometry

HO-1 expression was assessed in BM of 11 patients upon diagnosis. Then in patients in whom, after routine MRD staining, there was a sufficient material for additional staining, an intracellular HO-1 staining was performed (6 children at day 15, 3 children at day 33). In this purpose 10^6^ of bone marrow cells were stained with CD34-PE, CD45-PerCP, CD10-PE-Cy7, CD19-APC, CD38-AlexaFluor-700, CD20-APC H7, CD11a-BV510 (BD Biosciences, San Jose, CA, USA), lysed, fixed, and then permeabilized using a BD Intrasure Kit, according to the manufacturer’s instructions. After permeabilization step, cells were incubated with primary antibodies recognizing HO-1 (rabbit polyclonal, SPA 896, Enzo, Warszawa, Poland), washed twice, and stained with goat anti-rabbit secondary antibody conjugated with Alexa Fluor 488 (Life technologies, HITACHI, Tokyo, Japan). After two washing steps, samples were stained with Syto^®^41 (Thermo Fisher Scientific, Waltham, MA, USA) and analyzed using FACSCanto10 with FACSDiva Sorfware v 8.0.1 (Becton Dickinson Biosciences, San Jose, CA, USA).

### 4.10. Statistical Analysis

The results were analyzed using GraphPad Prism 7 software (GraphPad Software, San Diego, CA, USA). Relapse-free survival (RFS) curves were drawn with Kaplan–Meier methods, and differences in curves were analyzed using the log-rank (Mantel–Cox) test. To determine whether there is a significant difference between the observed frequencies in *HMOX1* polymorphisms the Fisher’s exact test was used. To compare the distribution between the two groups, the Mann–Whitney U test was used. To examine the influence of two different independent variables (a genotype and a risk group) on a number of CIN incidents, two-way analysis of variance was applied. Differences were considered statistically significant if the significance level (*p*) was less than 0.05.

## Data Availability

The data presented in this study are available on request from the corresponding author.

## Supplementary Materials

Supplementary Materials can be found at https://www.mdpi.com/1422-0067/22/3/988/s1. Supplementary Figure S1. Distribution of length polymorphisms in control and patients, presented as actual number of GT repeats. Supplementary Figure S2. Electropherograms showing A(-413)T SNP polymorphisms. Supplementary Figure S3. (A) A and T alleles frequencies in patient and control group. In ALL patients: 64 A alleles (54.2%) and 54 T alleles (45.8%); in control group: 26 A alleles(58.1%) and 26 T alleles (41.9%), (B) Comparison of percentage of patients and controls having at least one A allele to TT genotype. In ALL patients: AA and AT genotype: 47patients (79.7%), TT genotype: 12 patients (20.3%); in control group: AA and AT genotype: 24 individuals (77.4%), TT genotype: 7 individuals (22.6%), (C) Comparison ofpercentage of patients and controls with AA genotype. In ALL patients: AA genotype: 17patients (28.8%), AT and TT genotype: 42 patients (71.2%); in control group: AA genotype: 12 individuals (38.7%), AT and TT genotype: 19 individuals (61.3%). Supplementary Figure S4. (A) A and T alleles frequencies in control group and in patients stratified intoparticular risk group. In SR group: 21 A alleles (58.3%) and 15 T alleles (41,7%); IR group: 35 Aalleles (56.5%) and 27 T alleles (43.5%); HR group: 8 A alleles (40%) and 12 T alleles (60%). (B) Comparison of percentage of individuals having at least one A allele to TT genotype, alike incontrols and in patient stratified into particular risk groups. In SR group: AA and AT genotype: 15patients (83.3%), TT genotype: 3 patients (16.7%); in IR group: AA and AT genotype: 26 patients(83.9%), TT genotype: 5 patients (16.1%); in HR group: AA and AT genotype: 6 patients (60%), TT genotype: 4 patients (40%). (C) Comparison of percentage of individuals with AA genotype alike in controls and in patient stratified into particular risk groups. In SR group: AA genotype: 6 patients(33.3%), AT and TT genotype: 12 patients (66.6%); in IR group: AA genotype: 9 patients (29%), ATand TT genotype: 22 patients (71%); in HR group: AA genotype: 2 patients (20%), AT and TT genotype: 8 patients (80%).
