# Supplementary material for: Role of HMOX1 Promoter Genetic Variants in Chemoresistance and Chemotherapy Induced Neutropenia in Children with Acute Lymphoblastic Leukemia
Source: Int J Mol Sci. 2021 Jan 20;22(3):988. doi: 10.3390/ijms22030988 (PMC7863945; doi:10.3390/ijms22030988)
Supplement: Supplementary file 1 [file ijms-22-00988-s001.pdf]

As cut-off point criteria of short, medium and long alleles are inconsistent, we also demonstrated our data as raw values (Fig. S1). The investigation of A(-413)T SNP (Fig. S2) revealed no significant difference in T and A alleles frequencies in ALL patients compared to control group (Fig. S3A). We also compared proportion of AA, AT and TT genotypes in patients and controls and we did not find statistically significant difference (Fig. S3B). Similarly, the frequency of allele A that T between risk groups was not altered significantly, as well as proportion of patients with certain genotype AA, AT or TT (Fig. S4A, S4B). In HR group there were only slightly more patients displaying at least one T allele or displaying homozygous TT genotype. However, this result can be attributed to the effect of co-segregation of T allele with short alleles (which were predominant in HR group), than to regulatory properties of T allele per se. Since in contrast to unequivocal S allele distribution, the final distribution of T allele in patients was balanced by the presence of T alleles co-segregated with very long alleles in control group, the difference between the groups was lost.

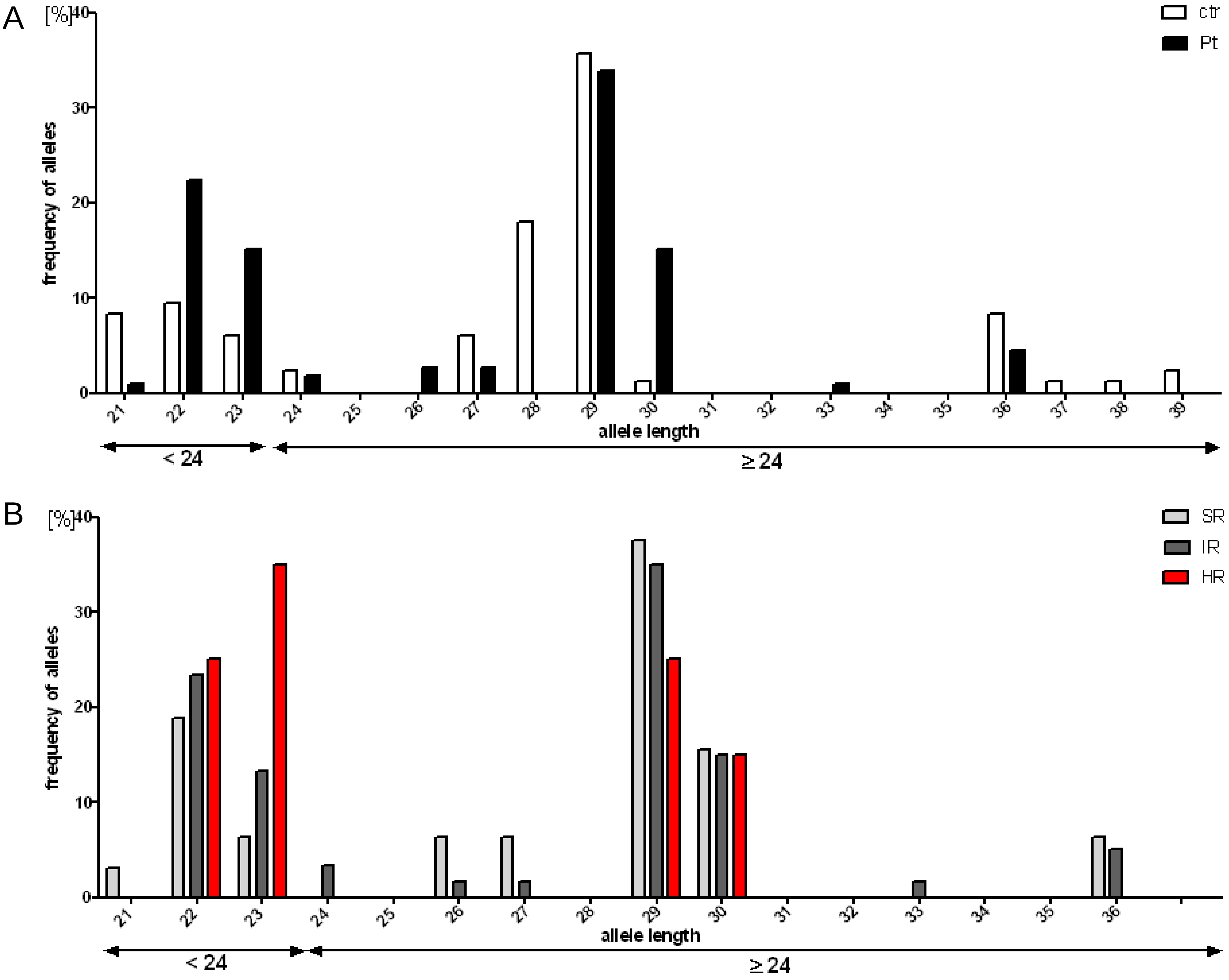

Sup. 1

Supplementary Fig. S1. Distribution of length polymorphisms in control and patients, presented as actual number of GT repeats

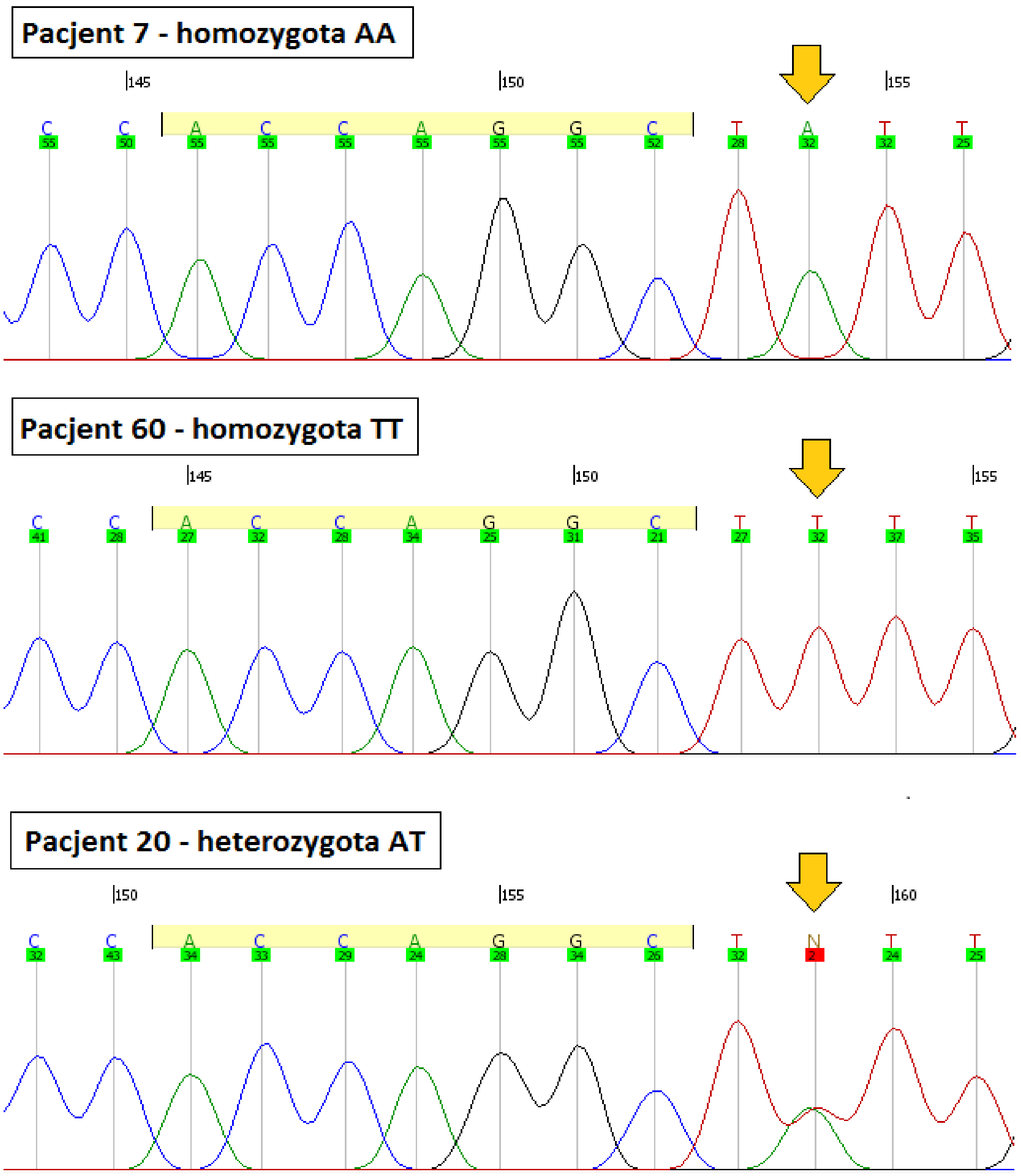

Sup. 2

Supplementary Fig. S2. Electropherograms showing A(-413)T SNP polymorphisms.

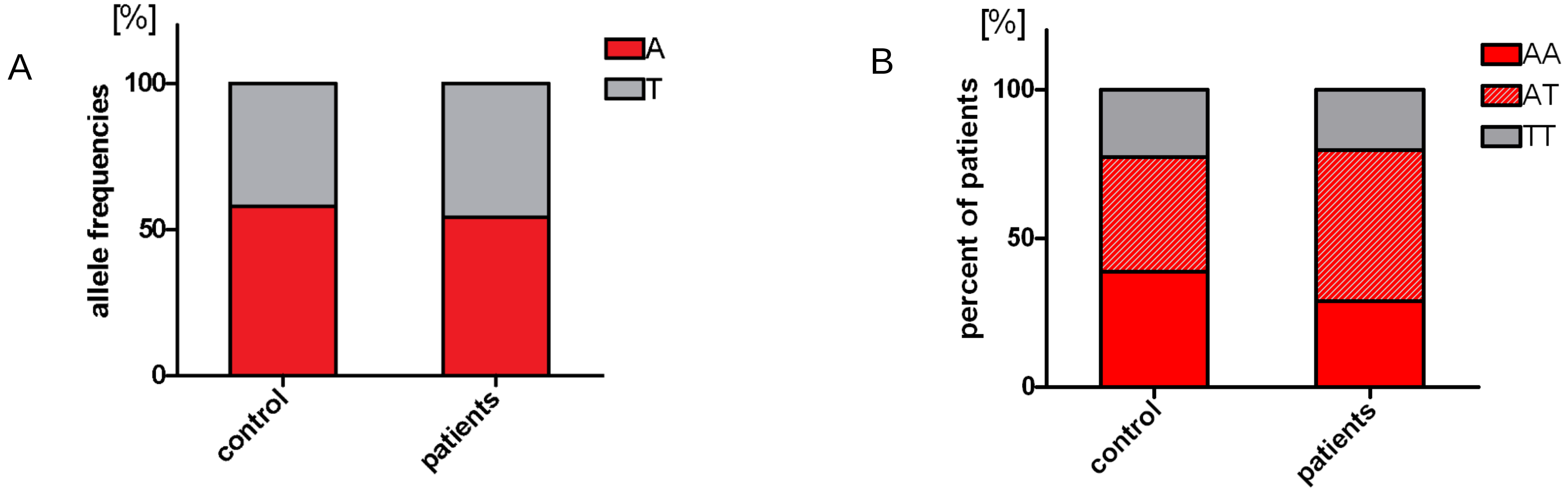

Sup. 3

Supplementary Fig. S3. A) A and T alleles frequencies in patient and control group. In ALL patients: 64 A alleles (54.2%) and 54 T alleles (45.8%); in control group: 26 A alleles (58.1%) and 26 T alleles (41.9%), B) Comparison of percentage of patients and controls having at least one A allele to TT genotype. In ALL patients: AA and AT genotype: 47 patients (79.7%), TT genotype: 12 patients (20.3%); in control group: AA and AT genotype: 24 individuals (77.4%), TT genotype: 7 individuals (22.6%), C) Comparison of percentage of patients and controls with AA genotype. In ALL patients: AA genotype: 17 patients (28.8%), AT and TT genotype: 42 patients (71.2%); in control group: AA genotype: 12 individuals (38.7%), AT and TT genotype: 19 individuals (61.3%).

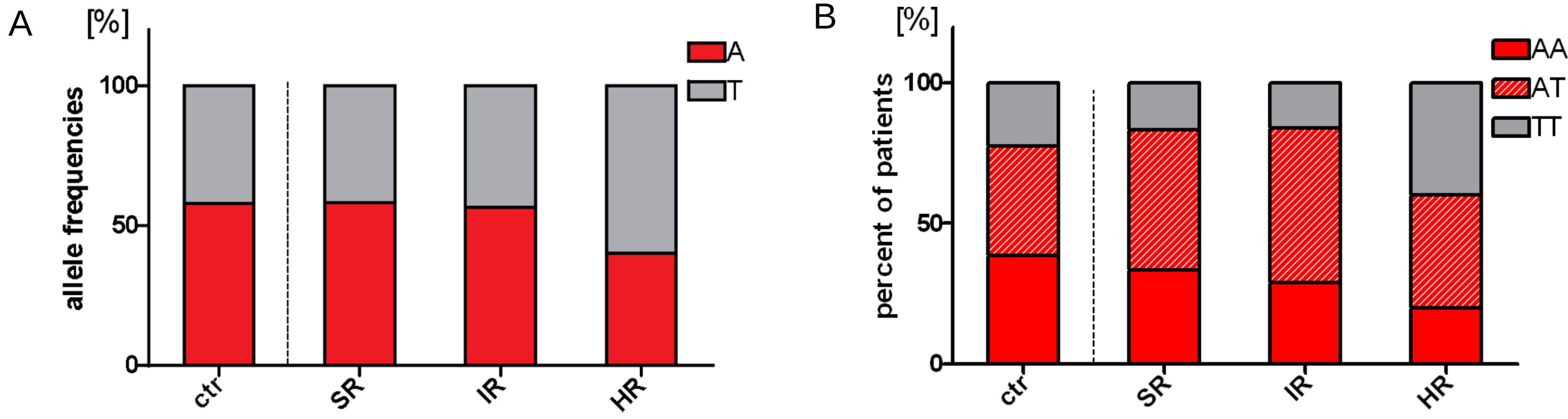

Sup. 4

Supplementary Fig. S4. A) A and T alleles frequencies in control group and in patients stratified into particular risk group. In SR group: 21 A alleles (58.3%) and 15 T alleles (41,7%); IR group: 35 A alleles (56.5%) and 27 T alleles (43.5%); HR group: 8 A alleles (40%) and 12 T alleles (60%). B) Comparison of percentage of individuals having at least one A allele to TT genotype, alike in controls and in patient stratified into particular risk groups. In SR group: AA and AT genotype: 15 patients (83.3%), TT genotype: 3 patients (16.7%); in IR group: AA and AT genotype: 26 patients (83.9%), TT genotype: 5 patients (16.1%); in HR group: AA and AT genotype: 6 patients (60%), TT genotype: 4 patients (40%). C) Comparison of percentage of individuals with AA genotype alike in controls and in patient stratified into particular risk groups. In SR group: AA genotype: 6 patients (33.3%), AT and TT genotype: 12 patients (66.6%); in IR group: AA genotype: 9 patients (29%), AT and TT genotype: 22 patients (71%); in HR group: AA genotype: 2 patients (20%), AT and TT genotype: 8 patients (80%).
